# Efficacy and safety of milk thistle preventive treatment of anti-tuberculosis drug-induced liver injury: A protocol for systematic review and meta-analysis

**DOI:** 10.1097/MD.0000000000023674

**Published:** 2020-12-24

**Authors:** Zhipeng Shi, Jing Wu, Qiang Yang, Hong Xia, Min Deng, Yuxia Yang

**Affiliations:** The People's Hospital of Dazu District Chongqing, Dazu, Chongqing, China.

**Keywords:** anti-tuberculosis drug-induced liver injury, milk thistle, preventive treatment, protocol, system evaluation

## Abstract

**Background:**

Tuberculosis is an infectious disease caused by mycobacterium tuberculosis. It may occur in multiple parts and organs of the patients body, and the lung is the most common. It is a major health threat worldwide. Hepatotoxicity is a common adverse reaction of commonly used clinical anti-tuberculosis drugs, as well as one of the important factors leading to poor prognosis of tuberculosis. Milk thistle is a traditional Chinese medicine extract derived from the mature fruit of Silybum marianum. Clinical practice shows that milk thistle has a good anti-liver injury effect and can be used to prevent anti-tuberculosis drug-induced liver injury. However, there is a lack of evidence-based medicine. The research carried out in this protocol is to systematically evaluate the efficacy and safety of milk thistle preventive treatment of anti-tuberculosis drug-induced liver injury, and to improve the evidence-based basis for clinical application of milk thistle in the preventive treatment of anti-tuberculosis drug-induced liver injury.

**Method:**

Computer search of English databases (PubMed, the Cochrane Library, Embase, Web of Science) and Chinese databases (CNKI, VIP, Wanfang, China Biology Medicine disc (CBMdisc)) was performed. From the establishment of database to October 2020, 2 researchers independently extracted and evaluated the data included in the randomized controlled clinical research of milk thistle preventive treatment of anti-tuberculosis drug-induced liver injury, and used RevMan5.3 software to conduct a meta-analysis of the included literature.

**Result:**

In this research, the efficacy and safety of milk thistle preventive treatment of anti-tuberculosis drug-induced liver injury were evaluated by indicators such as the incidence of liver injury, bilirubin levels, and liver enzyme levels.

**Conclusion:**

In this research, reliable evidence-based evidence for the clinical application of milk thistle in the preventive treatment of anti-tuberculosis drug-induced liver injury was provided.

**OSF Registration number:**

DOI: 10.17605/OSF.IO/VC3RM.

## 1 Introduction

Anti-tuberculosis drug-induced liver injury ranks first in reports on drug-induced liver injury, with an incidence of about 8% to 30%.^[1]^ The first-line anti-tuberculosis drugs commonly used in clinical practice, including rifampicin, isoniazid, pyrazinamide and streptomycin, induce varying degrees of liver injury, and liver injury will be severer after combined use.^[2]^ Anti-tuberculosis drug-induced liver injury may cause tuberculosis patients to deteriorate from asymptomatic liver enzyme to fulminant liver failure,^[3]^ anti-tuberculosis treatment may be discontinued in the clinic, dosage regimens are adjusted, drug compliance gets worse, and drug resistance becomes enhanced, etc.^[4]^ The prevention of anti-tuberculosis drug-induced liver injury has become a key factor that affects the treatment of tuberculosis.

Milk thistle is a traditional Chinese medicine extract derived from the mature fruit of the Compositae plant Silybum marianum. Milk thistle is mainly Class A of flavonoid. It consists of silibinin, silychristin, silidianin, isosilybin and flavone lignans, and unknown oxidized polyphenol compounds.^[5]^ A large number of clinical studies have shown that milk thistle can protect liver and prevent toxic metabolic liver injury from occurring through mechanisms such as anti-free radical activity, inhibition of lipid peroxidation, protection of liver cell membrane stability, inhibition of cytokines related to inflammation.^[6,7]^ Traditional medicine believes that milk thistle has the effects of heat-clearing and detoxifying and soothing liver-gallbladder,^[8]^ which can effectively promote the recovery of liver function.

At present, many in vitro and animal studies have shown that milk thistle is effective in preventing anti-tuberculosis drug-induced liver injury, with a low incidence of liver injury and fewer adverse reactions. However, the efficacy of milk thistle is still controversial. In addition, there are less clinical studies and several differences in research design and efficacy. Higher quality evidence-based evidence is needed to promote this therapy. Therefore, we conducted a meta-analysis to explore the effects of milk thistle on the degree of liver injury, liver enzyme levels and quality of life in tuberculosis patients who used anti-tuberculosis drugs, so as to provide the evidence-based basis for milk thistle preventive treatment of anti-tuberculosis drug-induced liver injury.

## 2 Method

### 2.1 Protocol register

This protocol of systematic review and meta-analysis has been drafted under the guidance of the preferred reporting items for systematic reviews and meta-analysis protocols (PRISMA-P). Moreover, the protocol has been registered on the open science framework (OSF) on November 4, 2020 (Registration number: DOI 10.17605/OSF.IO/VC3RM).

### 2.2 Ethics

Formal ethical approval is not necessary as the data cannot be individualized.

### 2.3 Eligibility criteria

#### 2.3.1 Types of studies

We collected all available randomized controlled trails (RCTs) on milk thistle preventive treatment of anti-tuberculosis drug-induced liver injury, which was not restricted by the published status.

#### 2.3.2 Research objects

Clinically diagnosed tuberculosis patients who had no previous history of liver disease and had normal liver function before treatment with unlimited nationality, race, age, gender, course of disease, and location of the disease. Those with cirrhosis or fatty liver, and other complications were excluded.

#### 2.3.3 Interventions

Patients were divided into trial group and control group and all of them were treated with standardized anti-tuberculosis treatment. Trial group was given milk thistle preventive liver protection treatment, while control group was given placebo treatment.

#### 2.3.4 Outcome indicators

1. Main outcome: Incidence of liver injury (serum AST or ALT > 2 times the upper limit of normal)
2. Secondary outcome: Glutamic-pyruvic transaminase level (alanine aminotransferase, ALT), glutamic oxalacetic transaminase level (aspartate transaminase, AST), total bilirubin level (total bilirubin level, TBil), incidence of adverse reactions

### 2.4 Exclusion criteria

1. Literature with repeatedly published research and the most complete and high-quality data;
2. Research literature with the research belonging to abstracts and conference papers, and the original data were not available;
3. Research with the data that had obvious logical errors and could not be processed after contacting the author;
4. Research with animal research, pharmacokinetics, or pharmacodynamic parameters;
5. Patients with liver injury caused by other reasons;
6. Research belonging to milk thistle preparation in vitro experiment.

### 2.5 Search strategy

Chinese search terms such as “milk thistle”, “anti-tuberculosis drugs” and “liver injury” were used to search in Chinese databases, including China National Knowledge Infrastructure (CNKI), VIP, Wanfang Data, CBMdisc; English search terms such as “silymarin”, milk thistle”, “tuberculosis”, “antitubercul”, “liver injury” were used to search in English databases, including PubMed, the Cochrane Library, EMBASE, Web of Science. The search time was from the establishment of database to October 2020, all domestic and foreign literatures on the milk thistle preventive treatment of anti-tuberculosis drug-induced liver injury were collected. Taking PubMed as an example, the search strategy is shown in Table 1.

**Table 1:** Search strategy in PubMed database.

Number	Search terms
#1	Silybin[MeSH]
#2	Silybin[Title/Abstract]
#3	Silybinin[Title/Abstract]
#4	Silymarin[Title/Abstract]
#5	Milk thistle[Title/Abstract]
#6	#1 OR #2 OR #3 OR #4 OR #5
#7	Tuberculosis[Mesh]
#8	Tuberculosis[Title/Abstract]
#9	Antitubercul[Title/Abstract]
#10	#7 OR #8 OR #9
#11	Chemical and drug induced liver injury[Mesh]
#12	Chemical and drug induced liver injury[Title/Abstract]
#13	Liver injury[Title/Abstract]
#14	Toxic Hepatitis[Title/Abstract]
#15	#11 OR #12 OR #13 OR #14
#16	#6 And #10 And #15

### 2.6 Data screening and extraction

**Figure 1:**
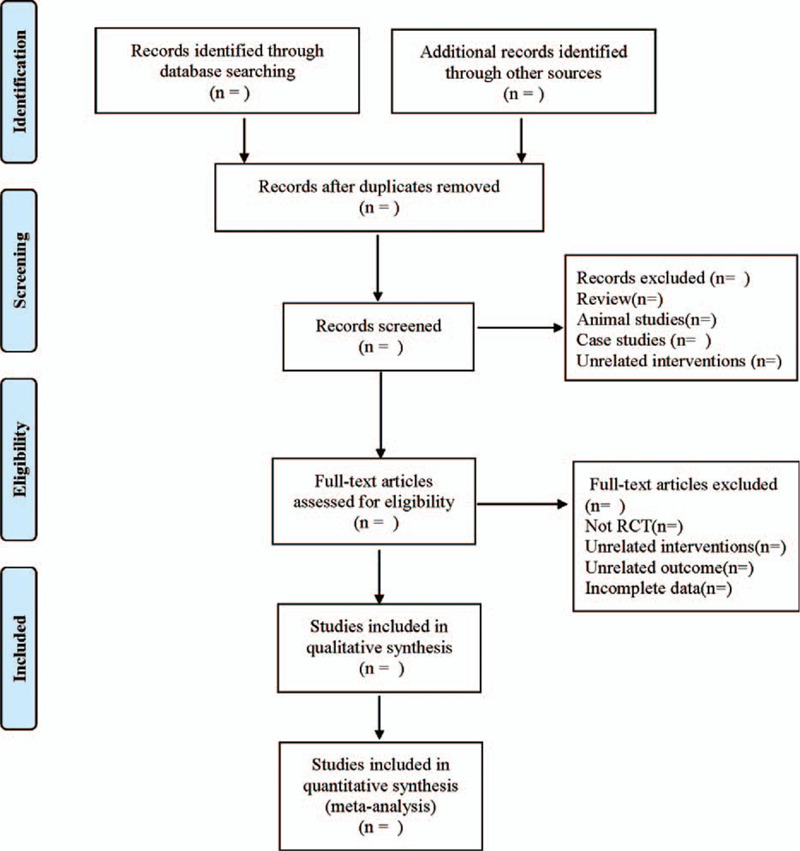
PRISMA flowchart.

The 2 researchers conducted preliminary screening by reading the titles and abstracts of the literature, and then conducted secondary screening by reading the full text. They referred to Cochrane Collaboration System Reviewer Manual Version 5.0 on the method of research selection, followed PRISMA flowchart, and independently screened the literature based on the above inclusion and exclusion criteria, and reviewed each other. In case of any divergence, a third party would participate in the discussion and negotiation. The information extracted from the literature included:

1. Clinical features (title, first author, publication year and month, sample size, sex ratio, average age, average course of disease);
2. Intervention measures: name, dose, frequency, and course of treatment of anti-tuberculosis drugs used in treatment group; name, dose, frequency and course of treatment of anti-tuberculosis drugs used in control group;
3. Evaluation factors of risk bias in randomized controlled studies;
4. Observation indicators. PRISMA flowchart would be used to show the research selection process (Fig. 1).

### 2.7 Literature quality evaluation

According to the bias risk evaluation tool for randomized controlled trials recommended in Cochrane System Reviewer Manual 5.1.0, the included literature was evaluated for bias risk. Specifically:

1. Whether to use random method;
2. Whether to achieve allocation hiding;
3. Whether to use blind method (single blind, double blind, triple blind);
4. Whether there were complete outcome data;
5. Whether there were selective report results;
6. Other biases.

The 2 researchers evaluated separately. If there was a disagreement, it would be resolved through negotiation. If no agreement was reached, it would be resolved by a third party.

### 2.8 Statistical analysis

#### 2.8.1 Data analysis and processing

RevMan5.3 software was used for meta-analysis. For dichotomous variables, relative risk (RR) was used for statistics. For continuous variables, when indicators of the tool and measurement unit were the same, weighted mean difference (WMD) was selected, as well as different tools or measurement units and standardized mean difference (SMD). All the above values were expressed with a 95% confidence interval (CI). Heterogeneity tests between the researches were judged by *I*^*2*^. *I*^*2*^ ≤ 50% indicated that there was no obvious heterogeneity between the researches, and the fixed effect model was used to calculate the combined effect size; *I*^*2*^ > 50 indicated that the heterogeneity between the researches was large. Subgroup analysis and sensitivity analysis were used to eliminate heterogeneity. If there was no obvious clinical heterogeneity between the researches, a random effect model was used for meta-analysis. If there was significant heterogeneity between the researches, meta-analysis should be abandoned if necessary, and only a descriptive analysis was conducted.

#### 2.8.2 Dealing with missing data

Contacting the corresponding authors of the included researches would be the solution to obtain missing or insufficient data for the main results, including emails or phone calls. If unavailable, researches with missing data should be deleted.

#### 2.8.3 Subgroup analysis

According to the age of patients, they could be divided into 4 subgroups: minors, young people, middle-aged people, and old people for subgroup analysis; subgroup analysis was conducted according to the location of tuberculosis, such as pulmonary tuberculosis, bone tuberculosis, etc.; and according to the types of anti-tuberculosis drugs and according to different courses of treatment.

#### 2.8.4 Sensitivity analysis

Sensitivity analysis was conducted to evaluate the impacts of sample size, methodological quality, research design and missing data, and to verify the effectiveness of review conclusions. The analysis was repeated after excluding low-quality researches.

#### 2.8.5 Assessment of reporting biases

A funnel chart could be used to test and observe symmetry. If the number of included researches was more than ten, Egger and Begg tests could be used for publication bias. *P* < .05 indicated that there was publication bias.

#### 2.8.6 Evidence quality evaluation

The Grading of Recommendations Assessment, Development, and Evaluation (GRADE) will be the tool to assess the quality of evidence. Bias risk, consistency, directness, precision, and publication bias will be assessed. And the quality of evidence will be rated as 4 levels: high, moderate, low, and very low.

## 3 Discussion

Tuberculosis is a chronic infectious disease caused by the bodys infection with mycobacterium tuberculosis. In accordance with estimates by the World Health Organization, about one-third of people in the world have been infected with pathogenic bacteria of tuberculosis, which is a major health threat worldwide.^[9]^ At present, the clinical application of anti-tuberculosis drugs has become mature, but it is still difficult to avoid its drug-induced liver injury. The mechanism of anti-tuberculosis drug-induced liver injury is not yet fully understood, but according to previous studies, anti-tuberculosis drugs can cause oxidative stress, lipid peroxidation, and depletion of glutathione reserves.^[9]^ For instance, isoniazide can cause liver injury through a variety of mechanisms, such as the action of toxic metabolites and the activation of oxidative stress pathways, leading to the production of reactive oxygen species, lipid peroxidation, etc.^[10]^; rifampicin can cause mixed types of liver injury and disordered expression levels of various proteins^[11]^; pyrazinamide can interfere with dehydrogenase, inhibit dehydrogenation to produce free radicals, and induce liver injury by inducing lipid peroxidation.^[12]^

Milk thistle is an extract of natural ingredients with stable structure, acceptable safety, and few reports of adverse reactions. The unique hospholipid solids of milk thistle make it have good bioavailability and absorbability, high patient tolerance, and less pain.^[13]^ Studies have shown that silymarin has a variety of liver protection mechanisms, including antioxidant effects, which can scavenge reactive oxide species and participate in the antioxidant function of glutathione^[14]^; anti-inflammatory effects can effectively reduce the level of inflammatory molecules^[15]^; biomembrane stabilizers and regulators can prevent toxic substances from entering liver cells; protein synthesis stimulators can promote liver regeneration^[16]^; stellate hepatocyte inhibitors can control changes in myofibroblasts and avoid collagen fibers deposition and liver cirrhosis.^[17]^

At present, many trials on the milk thistle preventive treatment of anti-tuberculosis drug-induced liver injury have been widely reported, but there is a lack of systematic and correct evaluation. Therefore, it is necessary to objectively evaluate milk thistle against anti-tuberculosis drug-induced liver injury through evidence-based medicine and promote milk thistle preventive treatment. However, this research also has certain limitations. The effects of milk thistle species, ingredients, and methods to improve bioavailability may vary. There are individual differences and other factors in the severity of the disease and anti-tuberculosis treatment plan of the samples included in the research, clinical heterogeneity cannot be ruled out.^[18]^ Meanwhile, only English and Chinese literatures are searched due to the limitation of language, researches in other languages may be ignored, and there may be a certain publication bias.

## Author contributions

**Data curation:** Zhipeng Shi, Jing Wu, Qiang Yang.

**Funding acquisition:** Yuxia Yang.

**Software:** Hong Xia, Min Deng.

**Supervision:** Yuxia Yang.

**Writing – original draft:** Zhipeng Shi, Jing Wu.

**Writing – review & editing:** Zhipeng Shi, Yuxia Yang.
